# Trends in Surgical Morbidity and Survival Outcomes for Radical Hysterectomy in West China: An 11-Year Retrospective Cohort Study

**DOI:** 10.3389/fonc.2022.836481

**Published:** 2022-02-10

**Authors:** Huining Jing, Ying Yang, Yinxia Liu, Peijun Zou, Zhengyu Li

**Affiliations:** ^1^ Department of Gynecology and Obstetrics, West China Second University Hospital, Sichuan University, Chengdu, China; ^2^ Key Laboratory of Birth Defects and Related Diseases of Women and Children, Sichuan University, Ministry of Education, Chengdu, China

**Keywords:** surgical morbidity, radical hysterectomy, cervical cancer, oncology, survival

## Abstract

**Objectives:**

To vertically analyze the trend of surgical approaches, demographics, surgical morbidity, and long-term survival outcomes of early-stage cervical cancer over the past 11 years and to determine whether there have been any significant changes.

**Methods:**

A total of 851 patients with consecutive International Federation of Gynecology and Obstetrics (FIGO) 2009 stage IA–IIA cervical cancer diagnosed between January 2008 and June 2018 at a single center in China were included in this retrospective study. Trends in the rate of minimally invasive surgery (MIS), demographics, surgical morbidities, and long-term survival outcomes were determined. We categorized patients into two groups according to their year of operation. The demographics, pathological factors, surgical morbidity, and long-term survival outcomes were compared between these two groups.

**Results:**

Regarding the surgical approach, there was a significant increase in the rate of laparoscopic radical hysterectomy (LRH) performed over the study period, from 7.8% in 2008 to 72.5% in 2018 (p < 0.0001). The mean age of patients who underwent abdominal radical hysterectomy (ARH) has increased slightly from 2008 to 2018, and those who underwent ARH in the second half of the study period (2014–2018) were significantly older (45.01 vs. 47.50 years; p = 0.001). The most impressive changes over the past 11 years have occurred in the surgical morbidity in both the ARH and LRH groups. The overall surgical morbidity decreased from 29.2% in 2008 to 11.9% in 2018, with an annual rate of 1.57%. The median estimated blood loss volume of the ARH group was 500 ml (range 50–2,000) in the first few years compared to 400 ml (30–2500) in the last few years of the study period (p < 0.0001), which in the LRH group was 350 ml (range 150–800) and 150 ml (range 5–1,000), respectively (p < 0.0001). Similarly, allogeneic blood transfusions and hospital stay have all decreased dramatically over time in both approaches. On the other hand, our study did not reveal any significant statistical changes in long-term survival outcomes over the follow-up period in either group.

**Conclusions:**

The findings of our study demonstrate that great progress in surgically managed cervical cancer has been made over the last decade in West China. Our retrospective study demonstrated that the year of operation does not appear to influence the long-term survival, but the surgical morbidity impressively decreased over the study period in both the ARH and LRH groups, which reflects that the higher hospital surgical volume for radical hysterectomy (RH) was not associated with lower survival outcomes but related to the reduction of surgical morbidity.

## Introduction

Globally, cervical cancer (CC) continues to be the fourth most common cancer among females, and 85% of new cases and 90% of deaths occur among people from socioeconomically weaker sections of society (1). China reported 98,900 new cases of CC and 30,500 deaths in 2015 (2). Previous guidelines (3) indicate that either open or minimally invasive surgery (MIS) is an acceptable surgical treatment to radical hysterectomy (RH) in patients with early-stage (IA2 to IIA) CC. These recommendations have led to the widespread use of the MIS approach in recent years after the implementation of laparoscopy during the 1990s. However, Ramirez et al. (4) reported a multicenter randomized controlled trial (RCT), namely, the LACC trial, which showed that MIS was associated with lower 4.5-year disease-free survival (DFS), progression-free survival (PFS), overall survival (OS), and disease-specific survival rates and a higher local recurrence rate than the laparotomic approach. Several multicenter retrospective studies from different countries have validated this finding (5–8). Reasons beyond these results are unclear. Some studies focused on the learning curves of the surgeons and discussed that the learning curves of MIS probably caused the decline in survival outcomes (9, 10).

However, the management of surgical patients involved the whole medical team, not only surgeons. We wondered whether team proficiency affects the survival outcomes. Previous studies involving women with early or locally advanced CC have demonstrated improvements in guideline compliance and outcomes at high-volume centers (11–16). However, there is currently no study involving the change of survival by years in a single center.

Therefore, this study aims to vertically analyze the trend of demographics, surgical approaches, and long-term survival outcomes of early-stage CC over the past 11 years, determine whether there have been any significant changes, and investigate the prognostic impact of different surgical year groups in patients with early CC undergoing RH in open and laparoscopic approaches.

## Method

### Study Design and Patient Enrollment

A total of 1,765 patients with consecutive International Federation of Gynecology and Obstetrics (FIGO) (2009) stage IA–IIA CC diagnosed between January 2008 and June 2018 at a single center in China were screened for eligibility in this retrospective study.

Inclusion criteria were the following: 1) patients underwent standard surgical treatment according to the National Comprehensive Cancer Network (NCCN) guidelines, a modified RH with pelvic lymphadenectomy (PLND) in stage IA1 with LVSI and stage IA2, and an RH with PLND with/without para-aortic lymphadenectomy in stage IB to IIA. 2) Patients have a histological subtype of squamous cell carcinoma, adenocarcinoma, or adenosquamous carcinoma regardless of histological grading.

Exclusion criteria were the following: 1) patients with incomplete follow-up data and 2) patients with severe fundamental diseases or pregnant.

Complete information, including demographics, clinical, and pathological information, was extracted from the Hospital Information System by two investigators. The demographics included age, menstruation, and body mass index (BMI); the clinical information included diagnosis, FIGO (2009) stage, surgical approach, date of surgery, hospital stay, duration of surgery, estimated blood loss, number of lymph node resected, and adjuvant treatment; the pathological information included histologic subtype, grading, lymphovascular space invasion (LVSI), stromal invasion depth, parametrial involvement, vaginal margin involvement, and lymph node metastasis. Recurrence was defined by clinical findings and imaging examinations, and all recurrences were confirmed by pathological analysis. This study was approved by the Institutional Ethics Committee of West China Second University Hospital (2019078), and all participants orally consented to the use of their medical records during telephone follow-up.

### Study Outcomes

The primary outcome of interest was PFS and OS in the whole study period and different phases. Secondary outcomes included the rate of the MIS approach versus the open approach for CC over the study period and trends in demographics and perioperative outcomes. Perioperative outcomes included blood transfusion, estimated blood loss, hospital stay, operation time, and postoperative complications, which are defined as those occurring during hospitalization, including urinary tract complications, paralytic ileus, incisional hernia, and deep venous thrombosis.

### Statistical Analysis

SPSS 23.0 software (IBM, Armonk, NY, USA) was used for statistical analysis. p < 0.05 was set to indicate statistical significance. Comparisons of continuous variables were conducted with parametric methods if assumptions of normal distribution were confirmed. Non-normally distributed variables and categorical data were compared between laparoscopic RH (LRH) and abdominal RH (ARH) groups with the use of non-parametric tests. Survival curves were generated by the Kaplan–Meier (K-M) method analyzed with log-rank test and multivariable Cox proportional hazards regression models. The enumeration data were analyzed *via* the chi-square test. The measurement data were analyzed *via* t-test and the Mann–Whitney U test between two groups while *via* ANOVA and the Kruskal–Wallis H test between multiple groups.

## Result

**Figure 1:**
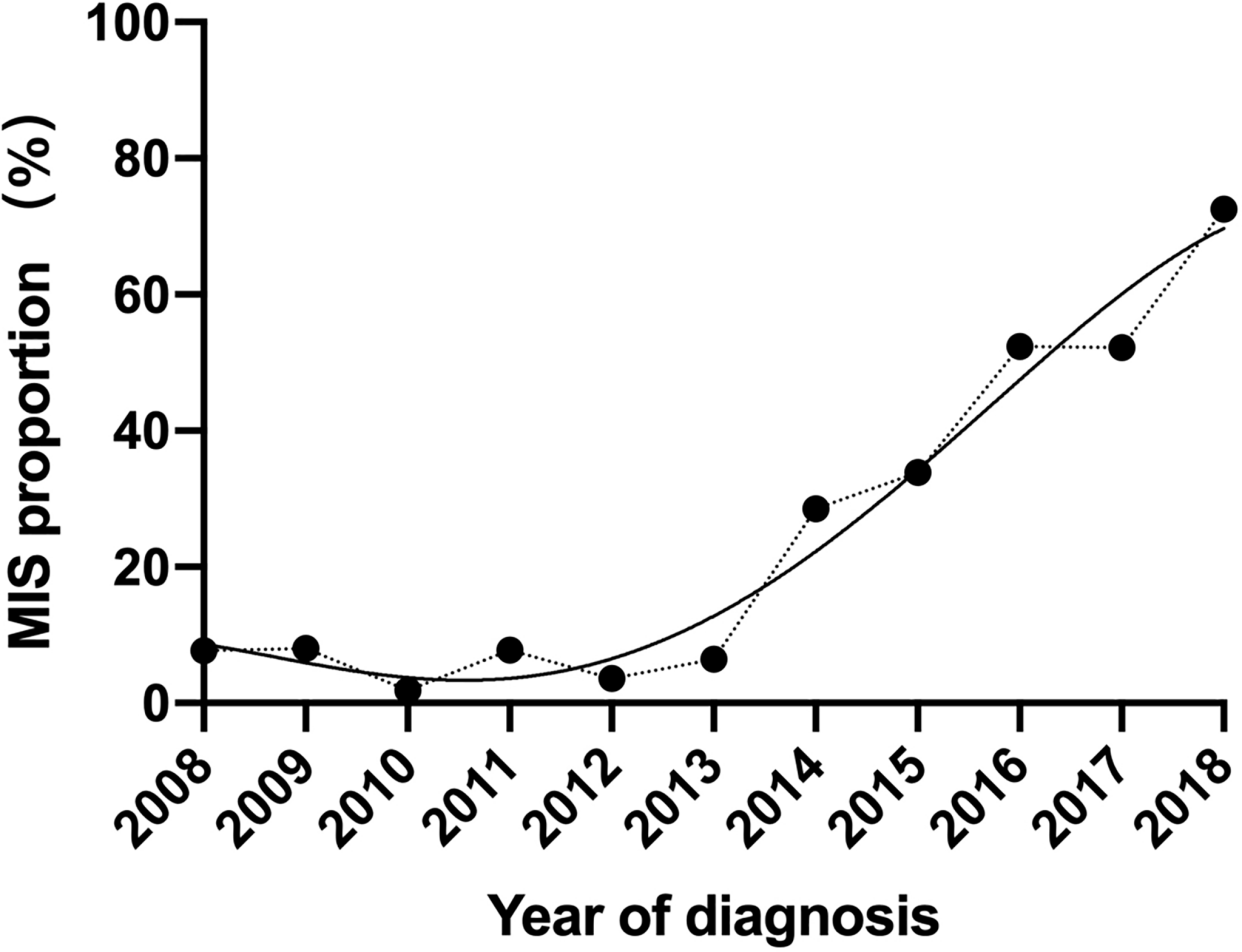
Crude rate of radical MIS for cervical cancer over study period.

After exclusions, a total of 851 women who were diagnosed with CC and had an RH (Querleu and Morrow type C2) in West China Second Hospital between January 1, 2008, and June 31, 2018, were included in this study. Among them, 581 (68.3%) had an abdominal approach, and 270 (31.7%) had a minimally invasive approach. All included operations were completed by five surgeons in our department. Regarding the surgical approach, there was a significant increase in the rate of LRH performed over the study period, from 7.8% in 2008 to 72.5% in 2018 (p < 0.0001). Our hospital began to carry out a large number of LRH operations in 2014. In the following years, it has increased at a stable rate, with an average annual increase of 13.2% of patients doing LRH (**Figure 1**).

**Figure 2:**
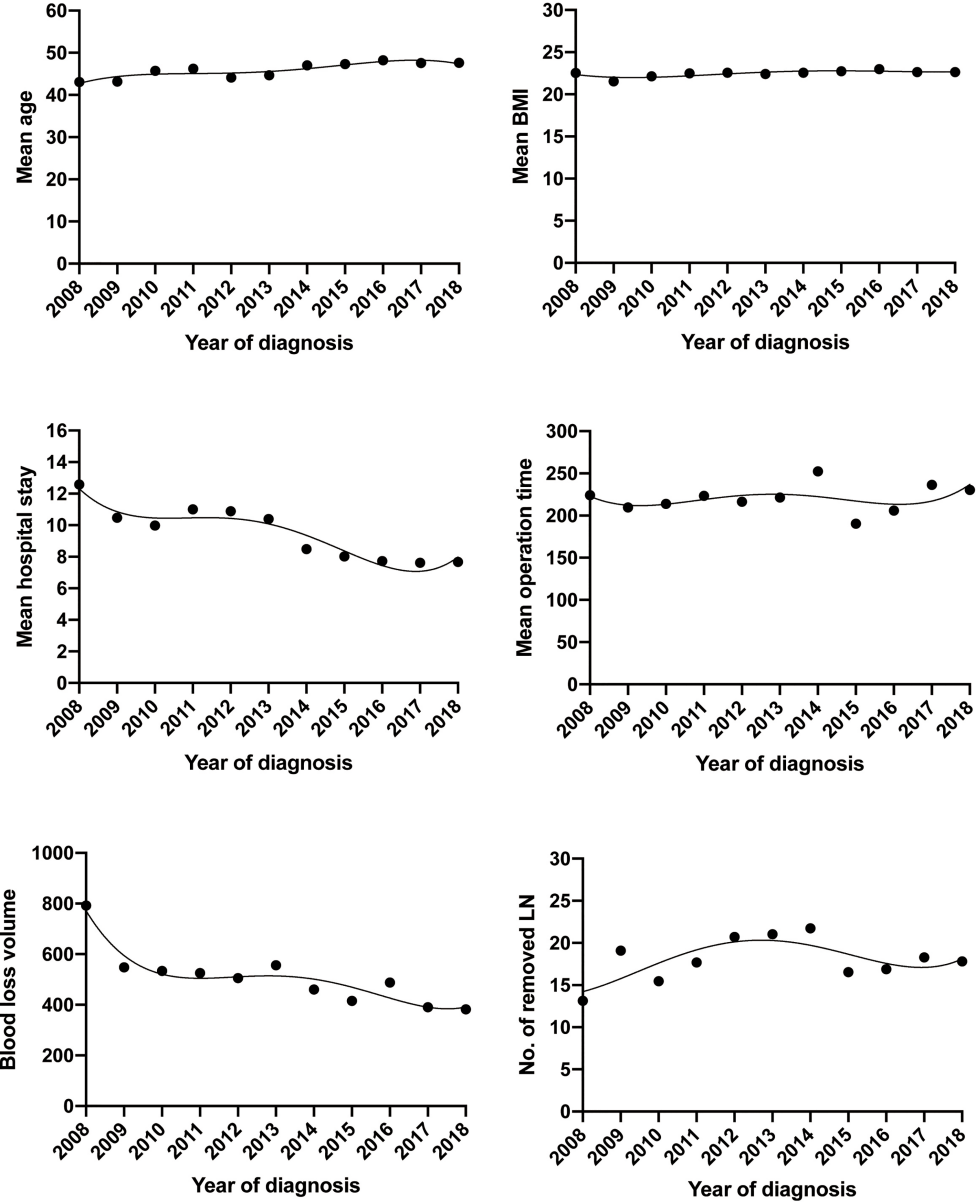
Trends of demographics and surgical morbidities with open approach of early-stage cervical cancer over the past 11 years.

As shown in **Table 1** and **Figure 2**, the mean age of patients who underwent ARH has increased slightly from 2008 to 2018, and those who underwent ARH in the second half of the study period (2014–2018) were significantly older (45.01 vs. 47.50 years; p = 0.001). The mean BMI of patients who underwent ARH had no upward or downward trend over time, which fluctuated in the range of 21.5–23 (p = 0.064). On the other hand, the age and the BMI of patients who underwent LRH had no statistically significant change over the past 11 years.

**Table 1:** Baseline characteristics in ARH and LRH groups.

Characteristics	ARH (N = 581)			LRH (N = 270)		
	2008–2013 (N = 295)	2014–2018 (N = 286)	p-Value	2008–2013 (N = 18)	2014–2018 (N = 252)	p-Value
Age (years), mean ± SD	45.01 ± 8.85	47.50 ± 8.76	**0.001**	46.61 ± 10.97	46.66 ± 9.11	0.982
BMI (kg/m^2^), mean ± SD	22.11 ± 3.74	22.64 ± 2.98	0.064	22.44 ± 3.52	23.13 ± 3.81	0.460
FIGO stage, N (%)			0.522			**0.032**
IA	21 (7.1)	14 (4.9)		5 (27.8)	24 (9.5)	
IB1	121 (41)	118 (41.3)		7 (38.9)	160 (63.5)	
IB2-IIA	153 (51.9)	154 (53.8)		6 (33.3)	68 (27.0)	
Grade, N (%)			**0.024**			0.778
G1/G2	35 (11.9)	55 (19.2)		6 (33.3)	64 (25.4)	
G3	240 (81.4)	206 (72)		10 (55.6)	151 (59.9)	
Gx	20 (6.8)	25 (8.7)		2 (11.1)	37 (14.7)	
Histology, N (%)			0.357			0.696
Squamous carcinoma	251 (85.1)	230 (80.4)		15 (83.3)	215 (85.3)	
Adenocarcinoma	30 (10.2)	34 (11.9)		3 (16.7)	29 (11.5)	
Adenosquamous carcinoma	13 (4.4)	21 (7.3)		0	6 (2.4)	
Other	1 (0.3)	1 (0.3)		0	2 (0.8)	
Stromal invasion			0.235			0.511
>1/2	178 (66.9)	151 (61.9)		10 (58.8)	99 (50.5)	
<1/2	88 (33.1)	93 (38.1)		7 (41.2)	97 (49.5)	
Positive lymph node metastasis	54 (22.0)	49 (19.5)	0.504	2 (18.2)	46 (23.2)	0.462
Parametrial invasion	25 (9.4)	46 (18.7)	**0.002**	4 (26.7)	20 (10.0)	0.069
Lymphovascular space invasion	133 (45.1)	105 (36.7)	0.536	5 (27.8)	96 (38.1)	0.382

ARH, abdominal radical hysterectomy; LRH, laparoscopic radical hysterectomy; BMI, body mass index; FIGO, International Federation of Gynecology and Obstetrics.

The bold values mean statistically significant (p< 0.05).

There was no significant shift in the proportion of patients with squamous cell carcinoma versus adenocarcinoma or the proportion of each FIGO stage in the ARH group over the past 11 years. Patients subjected to ARH have been diagnosed as more proportion of G1/G2 (11.9 vs. 19.2; p = 0.024) and more parametrial invasion (9.4 vs. 18.7; p = 0.002) in the second half of the study period, with no statistically significant change in the stromal invasion, incidence of pelvic lymph node metastases, or positive LVSI. Similarly, the pathological variables were analyzed in the LRH group, and the FIGO stage was the only variable that significantly changed over time (p = 0.032), with more stage IB1 in the last few years of the study period.

**Figure 3:**
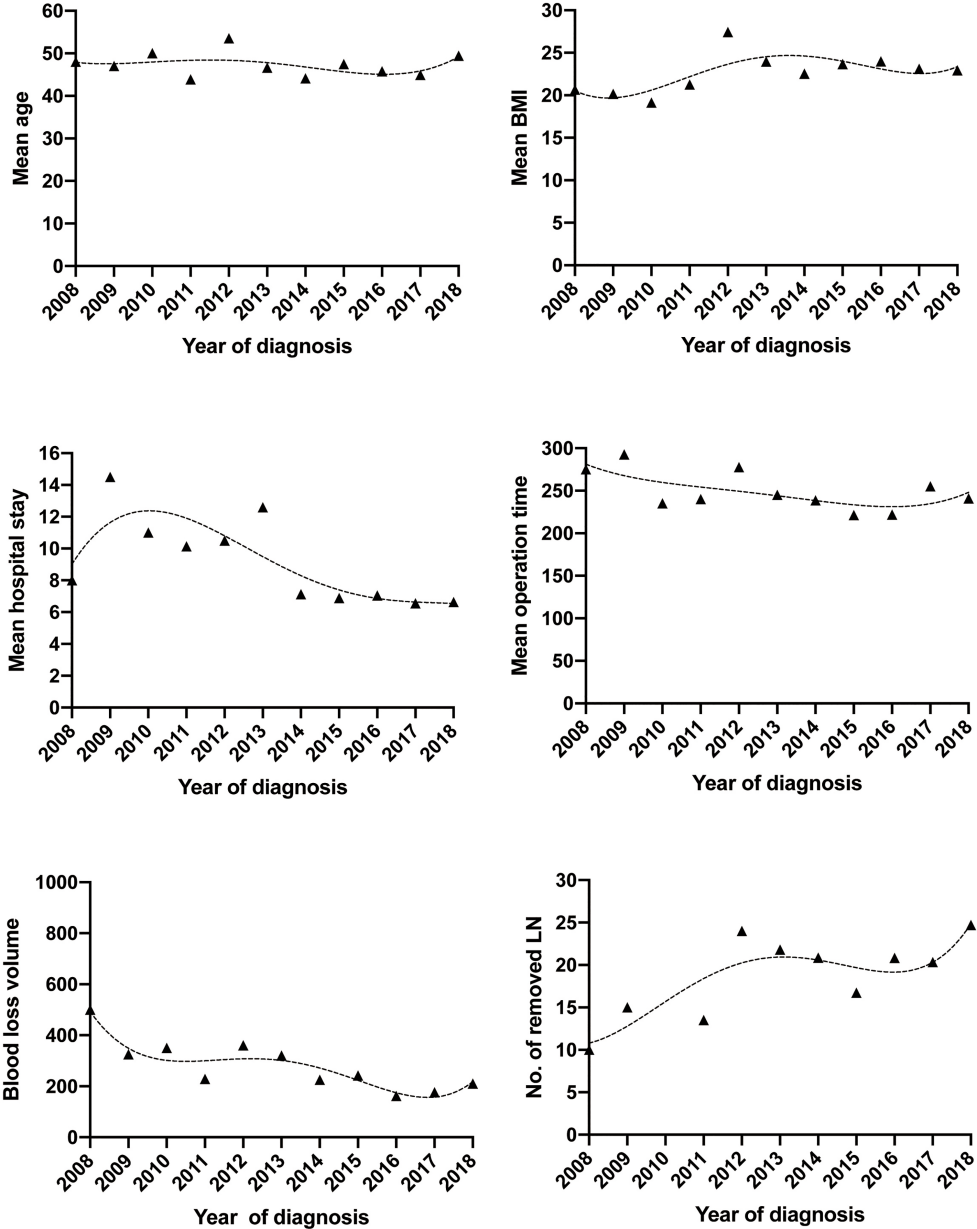
Trends of demographics and surgical morbidities with MIS approach of early-stage cervical cancer over the past 11 years. MIS, minimally invasive surgery.

The most impressive changes over the past 11 years, however, have occurred in the operative and postoperative short-term outcomes in both the ARH and LRH groups (**Table 2**). The median estimated blood loss volume of the ARH group was 500 ml (range 50–2,000) in the first few years compared to 400 ml (30–2,500) in the last few years of the study period (p < 0.0001), which in the LRH group was 350 ml (range 150–800) and 150 ml (range 5–1,000), respectively (p < 0.0001). Estimated blood loss volume, allogeneic blood transfusions, and hospital stay have all decreased dramatically in both approaches **Figure 3**. The median length of hospital stay of patients undergoing the open approach was 9 days (range 6–46) compared to 10 days (range 5–27) for the MIS approach in the first few years. By the last few years of the study period, the median length of hospital stay had significantly decreased to 7 days (range 3–24) following the open approach compared to 6 days (range 3–19) for MIS (p < 0.0001 in both approaches). The proportion of allogeneic blood transfusions of patients undergoing the open approach was 25.1% in the first few years compared to 6.2% in the last few years of the study period (p < 0.0001), which in the LRH group was 16.7% and 1.4%, respectively (p = 0.025). Although there were fluctuations, the median operation time of ARH remained stable over the past 11 years, floating around 220 min (3 h 40 min), whereas, in the LRH group projected, there was a downward trend, 275 min in 2008 compared to 240 min in 2018, but not statistically significant.

**Table 2:** Perioperative outcomes in ARH and LRH groups.

Characteristics	ARH (N = 581)			LRH (N = 270)		
	2008–2013 (N = 295)	2014–2018 (N = 286)	p-Value	2008–2013 (N = 18)	2014–2018 (N = 252)	p-Value
Blood transfusion	62 (25.1)	16 (6.2)	**<0.001**	2 (16.7)	3 (1.4)	**0.025**
Hospital stay, median (range, days)	9 (6–46)	7 (3–24)	**<0.001**	10 (5–27)	6 (3–19)	**<0.001**
Operation time, median (range, min)	210 (90–510)	200 (55–2500)	0.105	255 (150–360)	235 (65–450)	0.122
Estimated blood loss, median (range, ml)	500 (50–2000)	400 (30–2500)	**<0.001**	325 (100–800)	150 (5–1000)	**<0.001**
Postoperative complication						
No	209 (70.8)	252 (88.1)	**<0.001**	12 (66.7)	226 (89.7)	**<0.001**
Urinary tract complications	56 (19.0)	22 (7.7)		3 (16.7)	18 (7.2)	
Paralytic ileus	15 (5.1)	7 (2.5)		2 (11.1)	4 (1.5)	
incisional hernia	8 (2.7)	2 (0.7)		0 (0.0)	2 (0.8)	
Deep venous thrombosis	7 (2.4)	3 (1.0)		1 (5.6)	2 (0.8)	

ARH, abdominal radical hysterectomy; LRH, laparoscopic radical hysterectomy.

The bold values mean statistically significant (p< 0.05).

**Figure 4:**
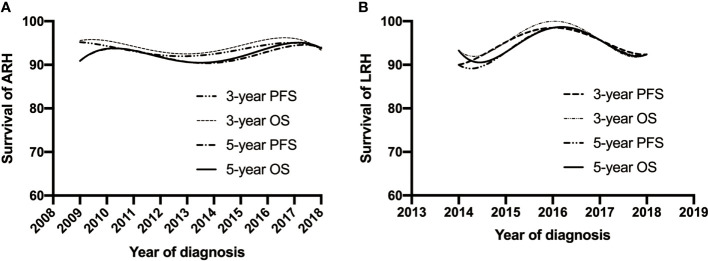
Trends of the OS and PFS adjusted for clinicopathological factors for patients with open approach **(A)** and MIS approach **(B)** of early-stage cervical cancer over the past 11 years. MIS, minimally invasive surgery; OS, overall survival; PFS, progression-free survival.

**Figure 5:**
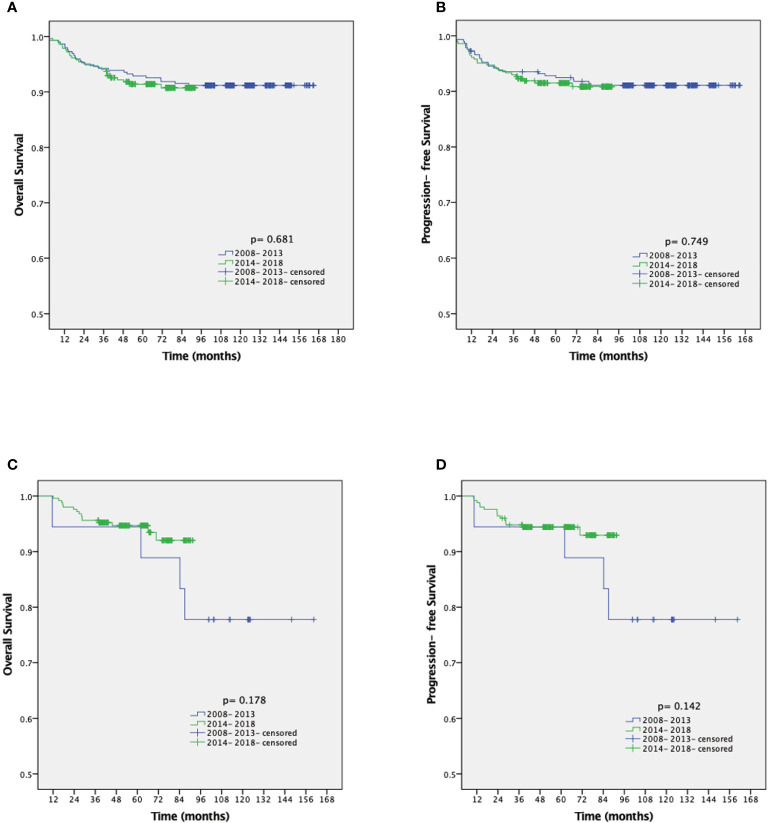
Kaplan-Meier analysis of the OS and PFS for patients with open approach stratified by year of diagnosis **(A, B)**. Kaplan-Meier analysis of the OS and PFS for patients with MIS approach stratified by year of diagnosis **(C, D)**. OS, overall survival; PFS, progression-free survival; MIS, minimally invasive surgery.

The median follow-up duration was 77.2 and 62.5 months in the ARH and LRH groups, respectively. The overall 3- and 5-year OS of the ARH group is 94.1% and 92.3%, respectively. The overall 3- and 5-year OS of the LRH group is 95.6% and 94.8%, respectively. When stratified by years of diagnosis, the chi-square test did not reveal any significant statistical changes of long-term survival outcomes over the follow-up period in either group (**Table 3** and **Figure 4**). Similarly, K-M survival analysis revealed no statistically significant differences between the 2008–2013 group and the 2014–2018 group in OS and PFS regardless of the surgical approaches (all p-value >0.05) (**Figure 5**).

**Table 3:** Survival outcomes of different years in ARH and LRH groups.

		2008	2009	2010	2011	2012	2013	2014	2015	2016	2017	2018	Total	p-Value
	Laparotomy cases	12	23	52	83	83	72	75	82	61	43	25	581	
ARH group	Three-year PFS (%)	100	95.7	94.2	90.4	94.3	94.4	92.0	91.5	91.8	100	92	93.3	0.763
	Three-year OS (%)	100	95.7	96.2	91.6	94.3	94.4	93.3	92.7	93.4	100	92.0	94.1	0.845
	Five-year PFS (%)	100	91.3	94.2	89.2	94.3	94.4	89.3	88.7	90.2	100	92.0	92.2	0.483
	Five-year OS (%)	100	91.3	94.2	89.2	94.3	94.4	89.3	89.0	91.8	100	92.0	92.3	0.489
	Laparoscopy cases	1	2	2	5	3	5	30	42	67	47	66	270	
LRH group	Three-year PFS (%)	–	–	–	–	–	–	90.0	95.2	98.5	95.7	92.4	94.8	0.429
	Three-year OS (%)	–	–	–	–	–	–	93.3	95.2	100	95.7	92.4	95.6	0.195
	Five-year PFS (%)	–	–	–	–	–	–	90.0	92.9	98.5	95.7	92.4	94.4	0.405
	Five-year OS (%)	–	–	–	–	–	–	93.3	92.9	98.5	95.7	92.4	94.8	0.540

ARH, abdominal radical hysterectomy; LRH, laparoscopic radical hysterectomy; OS, overall survival; PFS, progression-free survival.

## Discussion

In this research, we provided no differences in survival across the years despite the spread of the MIS approach to perform the RH; however, the surgical outcomes significantly improved over the years regardless of approach. Our study was based on a hypothesis of the surgical volume–outcome relationship that was originally reported in 1979 (17). The central concept of the volume–outcome relationship is that a larger surgical volume is associated with decreased surgical morbidity and mortality. Thus, we want to know whether the surgical morbidity and survival outcome of patients will be improved with the accumulation of the surgical volume and surgeons’ proficiency in our center.

The selection criteria for RH remained relatively stable over the research period, which allowed us to describe the change of patient demographics, pathology characteristics, surgical morbidity, and long-term survival outcomes with a small selection bias. These findings suggest that the year of operation does not appear to influence long-term survival. However, surgical morbidity has impressively decreased over the past 11 years in both the ARH and LRH groups.

The finding that surgical morbidity has decreased over the research period is not surprising. Many studies have indicated the same finding, which is almost indisputable (18, 19). The LRH for early CC has been utilized in developed countries since the early 1990s. However, in the underdeveloped areas of Western China, the introduction of this technology is about in the early 2010s. According to this study, our center, the most influential and technologically advanced tertiary hospital in this region of China, began to develop LRH rapidly in 2013–2014, and there were a small number of cases per year before 2014. Unlike LRH, ARH has been the standard approach for surgical treatment of early-stage CC for several decades. The hospital stay length and blood loss volume had still decreased slightly with the operation time remaining stable over the past 11 years. These changes have occurred with no improvement in the surgical procedure.

In our study, 5-year PFS and 5-year OS had no clear upward or downward trend over the study period, and the K-M survival curve showed no difference in the two groups divided by year. Whether the surgical volume affects survival remains controversial. Matsuo et al. (19) indicated that the hospital volume for RH may be a prognostic factor for early-stage CC and that high-volume centers are associated with decreased local recurrence risk and improved survival. A systematic review and meta-analysis suggested an association between high surgical volume and improved oncologic outcomes in MIS-RH for CC (20). However, Aviki et al. (21) indicated that there was no association between hospital volume and survival. A recent study also suggested that high-volume surgeon is not associated with better 5-year DFS and OS in cervical patients undergoing LRH (22).

The findings of our study demonstrate that great progress in surgically managed CC has been made over the last decade. The surgeon’s learning curve may be the explanation for the reduction in blood loss and blood transfusion. Previous studies have examined the learning curve in terms of the number of cases needed to obtain a relatively low hemorrhage volume, which is usually less than 50 cases (9, 23, 24). On the other hand, according to our data, blood loss has actually been decreasing slightly over the past 11 years. This may be explained by the assumption that surgeons are continuing to improve their surgical technique with time and experience after the early stage of the learning curve.

The main strength of this study is the large sample size. In addition, this is the first study to vertically analyze the trend of demographics, surgical approaches, surgical morbidity, and long-term survival outcomes of early-stage CC in West China. However, our study has several limitations. First, this is a retrospective study, and there may be unmeasured factors that confound the findings. Due to the nature of the retrospective study, it is difficult to achieve a balanced baseline between the two groups. In the ARH group, there is a higher rate of stage IB2-IIA (52.8% vs. 27.4%) and Grade 3 (76.8% vs. 59.6%). This is a subjective tendency based on experience that surgeons tend to choose laparotomy for patients with more severe conditions and laparoscopy for patients with lighter conditions. Second, tumor size data are not available in most cases, which may significantly impact the surgical outcome. Last, this is a single-center study, and the significant differences of institutional variables are unknown.

In conclusion, our retrospective study demonstrated that the year of operation does not appear to influence the long-term survival, but the surgical morbidity impressively decreased over the study period in both the ARH and LRH groups, which reflects that the higher hospital surgical volume for RH was not associated with lower survival outcomes but related to the reduction of surgical morbidity.

## Data Availability Statement

The datasets generated and/or analyzed during the current study are available from the corresponding author on reasonable request.

## Ethics Statement

The studies involving human participants were reviewed and approved by the Medical Ethics Committee of West China Second Hospital, Sichuan University. The patients/participants provided their written informed consent to participate in this study.

## Author Contributions

HJ and ZL contributed to the data collection and study design. YY, PZ, and YL contributed to the data analysis. All authors contributed to the data interpretation, manuscript preparation, editing, and review.

## Funding

This study was supported by grants from the Sichuan Youth Foundation of Science of Technology (grant number: 2015JQ0026).

## Conflict of Interest

The authors declare that the research was conducted in the absence of any commercial or financial relationships that could be construed as a potential conflict of interest.

## Publisher’s Note

All claims expressed in this article are solely those of the authors and do not necessarily represent those of their affiliated organizations, or those of the publisher, the editors and the reviewers. Any product that may be evaluated in this article, or claim that may be made by its manufacturer, is not guaranteed or endorsed by the publisher.
